# Cysteamine affects skeletal development and impairs motor behavior in zebrafish

**DOI:** 10.3389/fphar.2022.966710

**Published:** 2022-08-19

**Authors:** Chao Chen, Yongliang Zheng, Xue Li, Li Zhang, Kangyu Liu, Sujie Sun, Zilin Zhong, Hongmei Hu, Fasheng Liu, Guanghua Xiong, Xinjun Liao, Huiqiang Lu, Yanlong Bi, Jianjun Chen, Zigang Cao

**Affiliations:** ^1^ Birth Defects Group, Translational Research Institute of Brain and Brain-like Intelligence, Shanghai Fourth People’s Hospital, School of Medicine, Tongji University, Shanghai, China; ^2^ Department of Ophthalmology, Tongji Hospital, School of Medicine, Tongji University, Shanghai, China; ^3^ Department of Hematology, Affiliated Hospital of Jinggangshan University, Ji’an, JX, China; ^4^ Department of Hematology, The Second Affiliated Hospital of Xian Jiaotong University, Xi’an, China; ^5^ Department of Pediatrics, Shanghai Fourth People’s Hospital, School of Medicine, Tongji University, Shanghai, China; ^6^ Jiangxi Engineering Laboratory of Zebrafish Modeling and Drug Screening for Human Diseases, Jiangxi Key Laboratory of Developmental Biology of Organs, College of Life Sciences, Jinggangshan University, Ji’an, JX, China

**Keywords:** zebrafish, cysteamine, skeletal developmental defects, Notch signaling, oxidative stress

## Abstract

Cysteamine is a kind of feed additive commonly used in agricultural production. It is also the only targeted agent for the treatment of cystinosis, and there are some side effects in clinical applications. However, the potential skeletal toxicity remains to be further elucidated. In this study, a zebrafish model was for the first time utilized to synthetically appraise the skeletal developmental defects induced by cysteamine. The embryos were treated with 0.35, 0.70, and 1.05 mM cysteamine from 6 h post fertilization (hpf) to 72 hpf. Substantial skeletal alterations were manifested as shortened body length, chondropenia, and abnormal somite development. The results of spontaneous tail coiling at 24 hpf and locomotion at 120 hpf revealed that cysteamine decreased behavioral abilities. Moreover, the level of oxidative stress in the skeleton ascended after cysteamine exposure. Transcriptional examination showed that cysteamine upregulated the expression of osteoclast-related genes but did not affect osteoblast-related genes expression. Additionally, cysteamine exposure caused the downregulation of the Notch signaling and activating of Notch signaling partially attenuated skeletal defects. Collectively, our study suggests that cysteamine leads to skeletal developmental defects and reduces locomotion activity. This hazard may be associated with cysteamine-mediated inhibition of the Notch signaling and disorganization of notochordal cells due to oxidative stress and apoptosis.

## Graphical Abstract

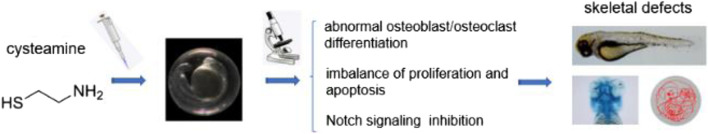

## Introduction

Cysteamine (CS), also known as β- mercaptoethylamine, is a component of Coenzyme A. It is a biologically active substance naturally existing in animals and plants. It contains active sulfhydryl groups and amino groups, participates in various physiological processes *in vivo*, and has multiple biological functions such as endocrine regulation, stress relief, immune regulation, and oxidation resistance (Naquet et al., 2020). Cysteamine alters the connection of the cysteine- cysteamine complex as an amino mercaptan and intercalator by inducing disulfide exchange. The reaction allows the lysosome to clear and transport the complex, and eventually to achieve the purpose of treating cystinosis. The most distinguishing feature of cystinosis is the lack of cystine lysosomal transporters, which leads to the systemic accumulation of cystine crystals and tissue impairment. The utilization of cysteamine significantly improved the long-term prognosis of patients. However, Besouw (Besouw et al., 2011) reported the toxicity and side effects of cysteamine on bone, skin, blood vessels, nerves, and muscles, which influenced approximately 1.5% of treated patients. Skin lesions and neurological symptoms improved with a reduction in the daily dose of cysteamine, with similar trends for bone lesions and muscle pain relief. The study of Bacchetta (Bacchetta et al., 2016) showed that the healing of stress fracture was delayed in the case of excessive cysteamine.

Furthermore, cysteamine is an ideal substance that can reduce the level of somatostatin (SS), promote digestion and metabolism, and accelerate animal growth. In recent years, as a feed additive, cysteamine has been extensively applied in animal husbandry and aquaculture (Tse et al., 2006; Du et al., 2012; Hu et al., 2016). Cysteamine hydrochloride considerably boosted serum growth hormone (GH) levels and promoted short-term growth augmentation in juvenile grass carp (Xiao and Lin, 2003). Cysteamine at a reasonable dose (1–3 mg/g) might increase the levels of GH and growth hormone receptor (GHR) mRNAs as well as the development of orange-spotted grouper (Li et al., 2013). However, most previous studies have focused on the pro-productive properties of cysteamine, and its toxic effects should be carefully evaluated and investigated further.

An early study confirmed that normal rats treated with cysteamine for 6 months developed severe skeletal deformities (kyphosis) as well as cardiovascular abnormalities (thoracic aortic dissection aneurysms) (Jayaraj, 1983). Likewise, cysteamine had a certain correlation with rat fetal cleft palate, kyphosis, intrauterine growth retardation, and intrauterine death (Beckman et al., 1998). *In vitro*, cysteamine induced cell death by inhibiting glutathione peroxidase and producing hydrogen peroxide (H_2_O_2_) in fibroblasts (Guha et al., 2019). Cysteamine administration induced glutathione depletion and nuclear transposition of apoptosis-inducing factor (AIF) in rats, resulting in apoptosis of duodenal epithelial cells (Cho et al., 2015). A study showed that the addition of cysteamine to buffalo semen diluent adversely affected semen quality after thawing, with decreased sperm motility and increased malondialdehyde concentrations found in samples treated with 5 mM cysteamine (Swami et al., 2017). These reports reveal the potential harm of cysteamine to cells and organisms. Nevertheless, the mechanisms involved in cysteamine toxicity have not been defined clearly, through which pathway it leads to skeletal damnification and skeletal dysplasia is worth exploring in depth.

Notch signaling pathway is a signal transduction system with a high degree of evolutionary conservation, which plays an important regulatory role in somite formation, osteoblast as well as osteoclast differentiation and maturation (Zanotti et al., 2008; Chen et al., 2014a). The mutation of key genes in Notch signaling pathway leads to the continuous activation or inhibition of Notch signal, resulting in a variety of skeletal system disorders. For example, mice with a simultaneous knockout of the Notch1 and Notch2 receptor genes exhibited a markable decrease in bone mass due to reduced osteoblast and enhanced bone resorption (Hilton et al., 2008). In human osteosarcoma cell lines, overexpression of Notch signaling genes (notch2, jagged1, and hey) was validated and inhibition of Notch signaling resulted in cell cycle arrest in G1 (Tanaka et al., 2009). Spondyloepiphyseal dysplasia (SD) is an autosomal recessive condition characterized by rib malformations, scoliosis, and the most frequently associated aberrations are mutations in the dll3 (Notch ligand gene) leading to a truncated or non-functional protein product (Sparrow et al., 2012). Moreover, Notch signaling pathway is closely related to oxidative stress. Notch signaling pathway regulates cell oxidative stress injury (OSI), and inhibition of Notch signaling pathway will affect cell activity, including cell proliferation and apoptosis (Androutsellis-Theotokis et al., 2006; Yang et al., 2013).

Zebrafish, as a model organism, is favored by many scientists because of the transparent embryos, large sample size, and easy feeding. It has been widely used in the toxicity detection of medical drugs and environmental pollutants (Mu et al., 2013; Li et al., 2016; Qiu et al., 2019; Xu et al., 2020). Zebrafish skeleton originates in three separate cell lineages, including cranial neural crest cells, axial mesoderm (somites), and lamina cells. These cell lines eventually differentiate into the craniofacial skeleton, mesoskeleton, and limb skeleton (Giffin et al., 2019). Mostly, the skeletal development of zebrafish starts with mesenchymal cell condensation, followed by differentiation into chondrocytes. Next, a cartilaginous template or mesenchymal primordium is formed, and later through endochondral ossification, a replacement of the perichondrium by hard bone containing osteoclast and osteoblast emerges. It culminates in the formation of mineralized bones, known as endochondral ossification (Yang, 2009). In zebrafish, Notch signaling is critically involved in skeletal development. Inhibition of Notch signaling tips the balance of skeletogenesis and resorption, causing related diseases (Henry et al., 2002; Giudicelli et al., 2007).

However, few studies have reported the toxicological effects of cysteamine on skeletal development in zebrafish. To fill this gap, we aim to gain insights into the adverse effects of cysteamine on embryonic skeletal development at the level of morpho-histology, behavior and gene expression, as well as the role of Notch signaling pathway in it. Using a zebrafish model, the current work provides significant data for a complete assessment of the skeletal toxicity of cysteamine.

## Materials and methods

### Reagents and chemicals

Cysteamine was purchased from Solarbio Technology Co., Ltd. (Beijing, China), and the drug was dissolved in embryo culture medium (salinity: 0.5%, CaCO_3_: 150 mg/L, pH: 7.0, conductivity: 500 μS/cm). Superoxide dismutase (SOD), catalase (CAT), malondialdehyde (MDA), reactive oxygen species (ROS) detection reagents, and Coomassie Blue were purchased from Nanjing Jiancheng Bioengineering Institute. RNAiso Plus was purchased from Takara Bio Inc. (Shiga, Japan). cDNA reverse transcription kit and qPCR kit were purchased from Beijing Quanjin Biological Co., Ltd. (Beijing, China). Sodium Valproate was purchased from MedchemExpress (New Jersey, United States).

### Maintenance of zebrafish and embryo collection

*Tg* (*twhh:GFP*) transgenic strains (marked Notochord cell), *Tg* (*tp1:GFP*) transgenic strains (marked Notch signaling pathway), and AB strains were obtained from China Zebrafish Resource Center. The zebrafish were culturing in the zebrafish breeding conditions as described previously (Cao et al., 2019). On the former night of mating, male and female adult zebrafish were placed in the breeding pond at a ratio of 1:1, and fertilized embryos were collected the next morning. The embryos were incubated in embryo culture medium at 28.5°C. At 24 h post-fertilization (hpf), the embryos were treated with 0.003% PTU (Sigma, United States) medium to suppress the growth of pigment. All animal experiments were conducted following the Laboratory Animal Welfare Guidelines of the Ministry of Science and Technology of the People’s Republic of China (2006) and with the approval of the Institutional Animal Care and Use Committee of Jinggangshan University.

### Chemical treatment

In the present study, 72 hpf was chosen as the effective time point to study cysteamine-induced skeletal toxicity, because zebrafish completed fully functional skeletal morphogenesis at 72 hpf. After exposure to different concentrations (0, 0.3, 0.6, 0.9, 1.2, 1.5, 1.8, 2.1, 2.4, 2.7, and 3.0 mM) of cysteamine, the mortality rate of zebrafish was registered at 24, 48, and 72 hpf. The most appropriate concentration was opted according to the mortality rate mentioned above. Well-developed *Tg* (*twhh:GFP*) and *Tg* (*tp1:GFP*) embryos at 6 hpf were assigned to six-well plates of 20 embryos per well and exposed to 0.35,0.70, and 1.05 mM cysteamine (final concentration) up to 72 hpf, respectively, while the control group was treated with culture medium only. Cysteamine exposure solution was changed every 24 h. In the sodium valproate (SV) rescue experiment, 6 hpf zebrafish embryos were cultured with a mixture of 1.05 mM cysteamine and 60 µM sodium valproate and processed to 72 hpf. The embryo medium treatment group and the 1.05 mM cysteamine treatment group served as controls. The experiment was conducted three times at least.

### Analysis of zebrafish skeletal morphology changes

The development of cartilage at 72 hpf and mineralized structure at 120 hpf were evaluated using alcian blue and calcein staining procedures, respectively (Félix et al., 2021). To assess cranial cartilage, the larvae were fixed overnight in 4% PFA at 4°C after euthanasia (immersion in culture medium containing 4% tricaine), followed by dehydrating and staining with 0.02% alcian blue in 70% ethanol and 60 mM MgCl_2_ overnight. Larval tissues were bleached in 3% H_2_O_2_ and 2% KOH to further remove the pigment to minimize the background after staining and digested in 0.05% PBS trypsin. Gradient concentrations of glycerol/KOH (20%/0.25%, 50%/0.25%, and 80%/0.1%) were used to further remove the excess tissue. ImageJ was used to quantify the cranial cartilage area (blue area) after alcian blue staining, which was normalized relative to the results of the control group. To analyze the mineralized structure, the larvae were soaked in fluorescent calcein solution (0.2%, pH 7.2) for 10 min in a room temperature, dark area. A stereo microscope (Zeiss Discovery. V20, Leica M205 FA) was used to capture pictures. All settings were consistent across within-group comparisons.

### Analysis of spontaneous movement and behavior

A stereo microscope (Zeiss Discovery. V20, Leica M205 FA) mounted with a high-resolution camera was used to record tail coilings of embryos for 1 min at 24 hpf. Twenty embryos in each group were selected randomly. After treating with drugs in each group till 72 hpf, the non-deformed larvae were transferred to a 24-well plate with a single larva in each well. When larvae were raised to the fifth day, the Noldus DanioVision system was used to monitor the effect of cysteamine on the behavior of zebrafish with skeleton fully developed. Before monitoring, zebrafish acclimated at 28°C for 20 min. The swimming patterns of zebrafish under light-to-dark transition (1-min dark, 1-min light, 10 cycles) were recorded. The heatmaps and trajectory plots were finally generated from the recordings, and the total moving distance, average moving speed, moving time, mobility, sinuosity, and turn angle were counted. The experiment was repeated at least 3 times.

### Analysis of oxidative stress

After cysteamine exposure till 72 hpf, 80 zebrafish embryos were anesthetized at low temperature in each group and washed 3 times with 1 × PBS, 5 min per time. Then they were homogenized with normal saline. After centrifuging × 10,000 g for 10 min at 4°C, the supernatant was used for enzyme activity detection. The total protein content of each group was determined with BCA method. SpectraMax^®^ iD3 Multi-Function Microplate Detector was used to obtain absorbance. SOD was measured at 550 nm by the inhibition of nitrite formation. CAT was measured by the decomposition of hydrogen peroxide at 405 nm and MDA was measured at 532 nm by the reaction with thiobarbituric acid. In the present study, SOD, CAT, and MDA were measured by specific kits. Zebrafish embryos at 72 hpf were treated for 30 min at room temperature in a culture medium containing 10 mg/ml dichloro dihydro fluorescein diacetate (DFCH-DA). After rinsing and anesthesia, the distribution map of ROS was taken by a three-dimensional microscope (Leica, Germany). All procedures were implemented under the recommendations of the manufacturer (Nanjing Jiancheng Bioengineering Institute, China). More detailed information on the methodology can be found in the Supplementary Material. SOD, CAT, and MDA were normalized by the total protein content of each group and ROS distribution was normalized by the mean fluorescence intensity relative to the control group. The experiment was repeated 3 times.

### Histological analysis

Zebrafish larvae were fixed at 4°C with 4% PFA overnight. They were dehydrated in gradient concentration of ethanol, transparentized with dimethyl benzene, impregnated in paraffin, and sectioned with a microtome (Leica, Germany). Paraffin sections at 7 µm thickness were dried at 37°C and then stained with HE. Histopathological evaluation was performed by observing and image capturing under a microscope (Zeiss, Germany). The proportion of each group (*n* = 10) showing abnormal aggregation of notochord cells was counted.

### Gene transcription analysis

To evaluate exposure to cysteamine on the transcriptional level, qPCR was performed to measure the gene expression related to skeletal development. 1 µg total RNA extracted by RNAiso Plus (Takara) was used for cDNA reverse transcription. After the cDNA was obtained, a CFX 96 system (Bio-Rad, Hercules, CA, United States) was used to detect transcriptional levels of genes. Housekeeping gene (ef-1α) was used as an internal control, and the result was displayed by 2^−△△Ct^ equation. Related primers for osteoblast marker genes (runx2a, bmp4, and col1a1), osteoclast marker genes (nfatc1, fosab, ctsk, and acp5b), Notch signaling pathway genes (notch1a, notch3, dlb, dll4, jag1a, jag2b, rbpjb, her6, and hey), cell cycle key genes (ccne1, cdk2, cdk6, and ccnd1), apoptosis genes (caspase3, p53, and bax) and anti-apoptotic genes (bcl2) were described before (Shi et al., 2020; Han et al., 2021; Molagoda et al., 2021).

### Statistical analysis

All the data were expressed as mean ± standard deviation. Before conducting statistical analysis, all data were examined for normality and variance homogeneity using the D’ Agostino-Pearson test. The differences between groups were analyzed by one-way ANOVA and Dunnett’s multiple comparisons tests. Statistical significance was set at *p < 0.05, **p < 0.01, ***p < 0.001 compared to the blank control group if not otherwise stated (Huang et al., 2021a).

## Results

### Cysteamine induced skeletal developmental defects in zebrafish embryos

**FIGURE 1:**
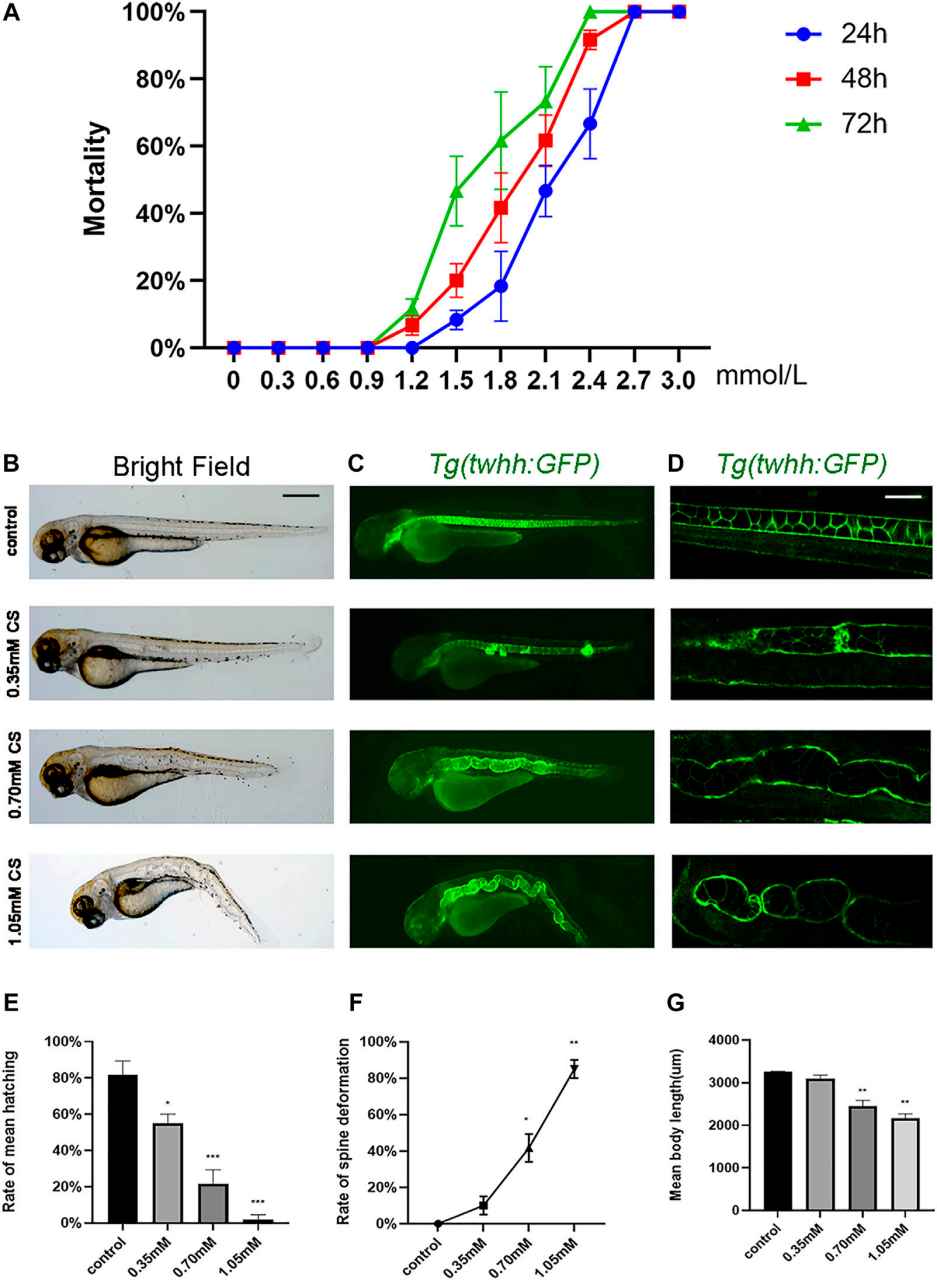
The phenotype of skeletal development defects in embryos after cysteamine exposure. **(A)** The cumulative mortality rate of zebrafish exposed from 0 to 3.0 mM cysteamine at 24, 48 and 72 hpf. **(B–D)** Microscopic imaging of Tg (twhh: GFP) zebrafish larvae exposed to 0.35, 0.70, and 1.05 mM cysteamine at 72 hpf. **(E)** The hatching rate of zebrafish exposed to 0.35, 0.70, and 1.05 mM cysteamine at 72 hpf. **(F)** The deformation rate of zebrafish exposed to 0.35, 0.70, and 1.05 mM cysteamine at 72 hpf. **(G)** The body length of zebrafish larvae exposed to 0.35, 0.70, and 1.05 mM cysteamine at 72 hpf. Scale bars: 100 μ **(B,C)**, 50 μm **(D)**. *p < 0.05, **p < 0.01, ***p < 0.001, mean ± S.D.

To determine the optimal exposure concentration, cysteamine concentrations from 0 to 3.0 mM were used to calculate the relative curve. At 72 hpf, larval death occurred in the 1.2 mM group, and the cumulative mortality rate increased sharply at 1.5 mM and reached 100% at 2.4 mM (Figure 1A). The LC_50_ values of cysteamine for zebrafish at 24, 48, and 72 hpf were 2.135, 1.878, and 1.614 mM, respectively. For subsequent experiments, sublethal concentrations (0.35, 0.70, and 1.05 mM cysteamine induced mild, moderate and severe skeletal defects, respectively) were chosen as exposure gradients.

Zebrafish embryos were continuously exposed to different concentrations (0.35, 0.70, and 1.05 mM) of cysteamine from 6 hpf to 72 hpf. Zebrafish larvae showed multiple abnormalities, with phenotypes of spinal curvature and disappearance of somite under confocal microscopy (Figures 1B–D). Larvae body length was shortened in 0.70 mM (2,451.47 ± 110.70, *p* = 0.008) and 1.05 mM (2,165.28 ± 87.20, *p* = 0.002) compared with the control group (3,254.54 ± 13.19) (Figure 1G). As the exposure concentration increased, the hatching rate decreased (*p* < 0.001, Figure 1E) and the deformation rate ascended in zebrafish embryos (*p* < 0.001, Figure 1F). The deformation rate caused by 1.05 mM cysteamine was 85% (Figure 1F).

**FIGURE 2:**
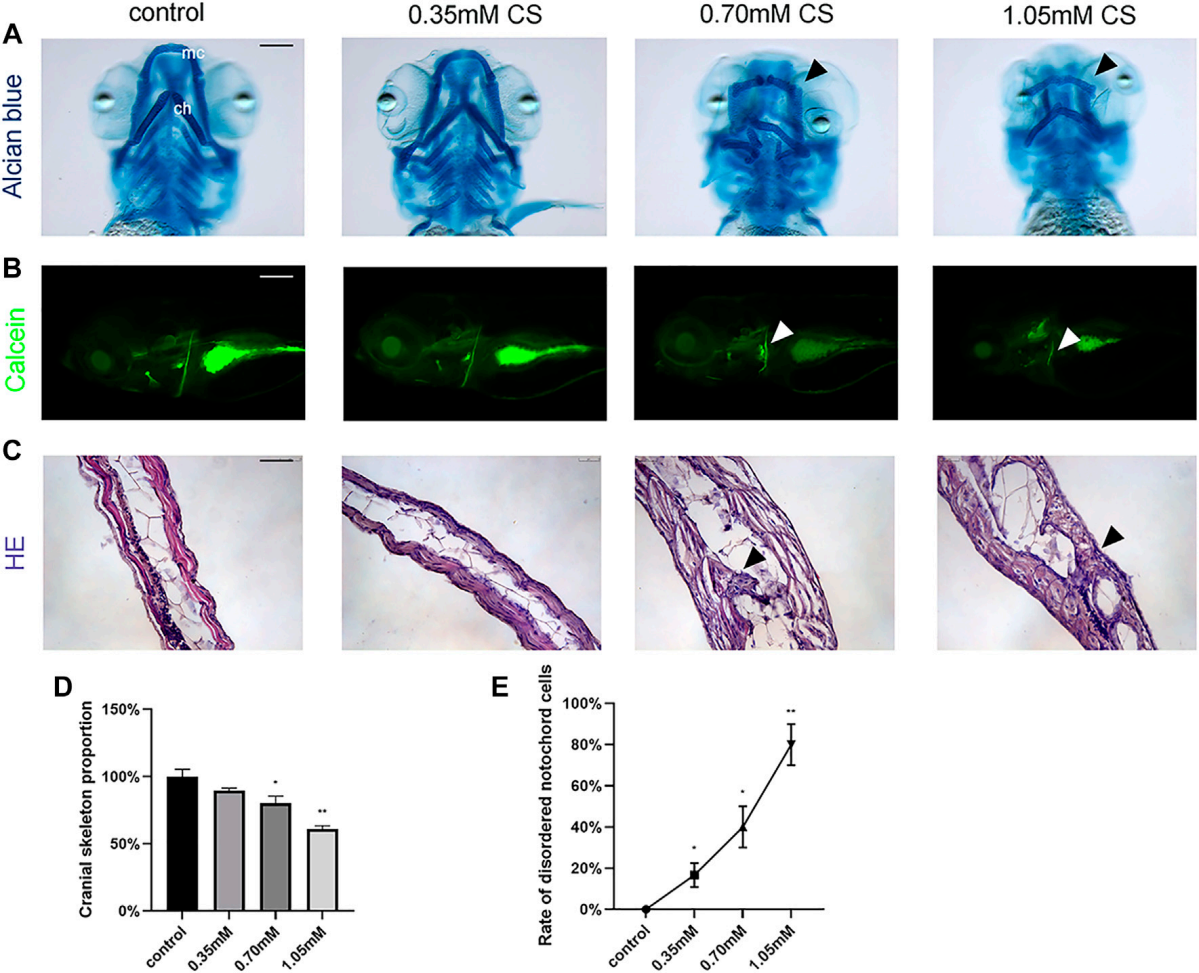
Skeletal staining of embryos after exposure to cysteamine. **(A)** Alcian blue staining of zebrafish larvae craniofacial skeleton exposed to 0.35, 0.70, and 1.05 mM cysteamine at 72 hpf. **(B)** Calcein staining of the whole-mount skeleton of zebrafish larvae exposed to 0.35, 0.70, and 1.05 mM cysteamine at 120 hpf. Arrows indicated abnormal craniofacial skeletal structure and chondropenia. mc: meckel’s cartilage, ch: ceratohyals. **(C)** HE staining of the skeleton of zebrafish larvae exposed to 0.35, 0.70, and 1.05 mM cysteamine at 72 hpf. Arrows indicated disordered accumulation of notochord cells and abnormal tissue vacuoles. **(D)** The craniofacial skeleton area of zebrafish larvae exposed to 0.35, 0.70, and 1.05 mM cysteamineat 72 hpf. **(E)** Rate of disordered notochord cells in zebrafish larvae exposed to 0.35, 0.70, and 1.05 mM cysteamine at 72 hpf. Scale bars: 50 μm **(A,C)**, 100 μm **(B)**. *p < 0.05, **p < 0.01, ***p < 0.001, mean ± S.D.

To determine the effect of cysteamine on cartilage and skeletal mineralization, alcian blue dye and calcein dye were performed at 72 hpf and 120 hpf, respectively. Alcian blue staining demonstrated abnormal craniofacial skeletal structure and chondropenia in zebrafish (Figure 2A). Quantitative analysis showed significant cartilage loss in the 0.70 mM (0.60 ± 0.04, *p* = 0.010) and 1.05 mM (0.46 ± 0.02, *p* = 0.002) groups compared to the control group (1.00 ± 0.04) (Figure 2D). In addition, defective chondrogenesis with respect to architecture and skeletal structure shape was observed in the 0.70 and 1.05 mM groups (shown by arrows). Specifically, the lengths and angles of meckel’s cartilage (mc) and ceratohyals (ch) were distorted. Calcein staining at 120 hpf in zebrafish from treatment group showed a reduction in mineralized structures, suggesting that the effects of cysteamine on skeletal development might be persistent and irreversible (Figure 2B).

The results of HE staining showed abnormal morphology of skeletal structure in the high-concentration group. Disordered accumulation of notochord cells, as well as significantly abnormal tissue vacuoles, were observed in the experiment group (Figure 2C). The percentage of occurrence of this phenomenon tended to increase with increasing exposure concentration (*p* < 0.001, Figure 2E). The results showed that cysteamine induced the disruption of zebrafish skeletal structure and led to skeletal developmental defects.

### Cysteamine induced unusual spontaneous movement and behavior

**FIGURE 3:**
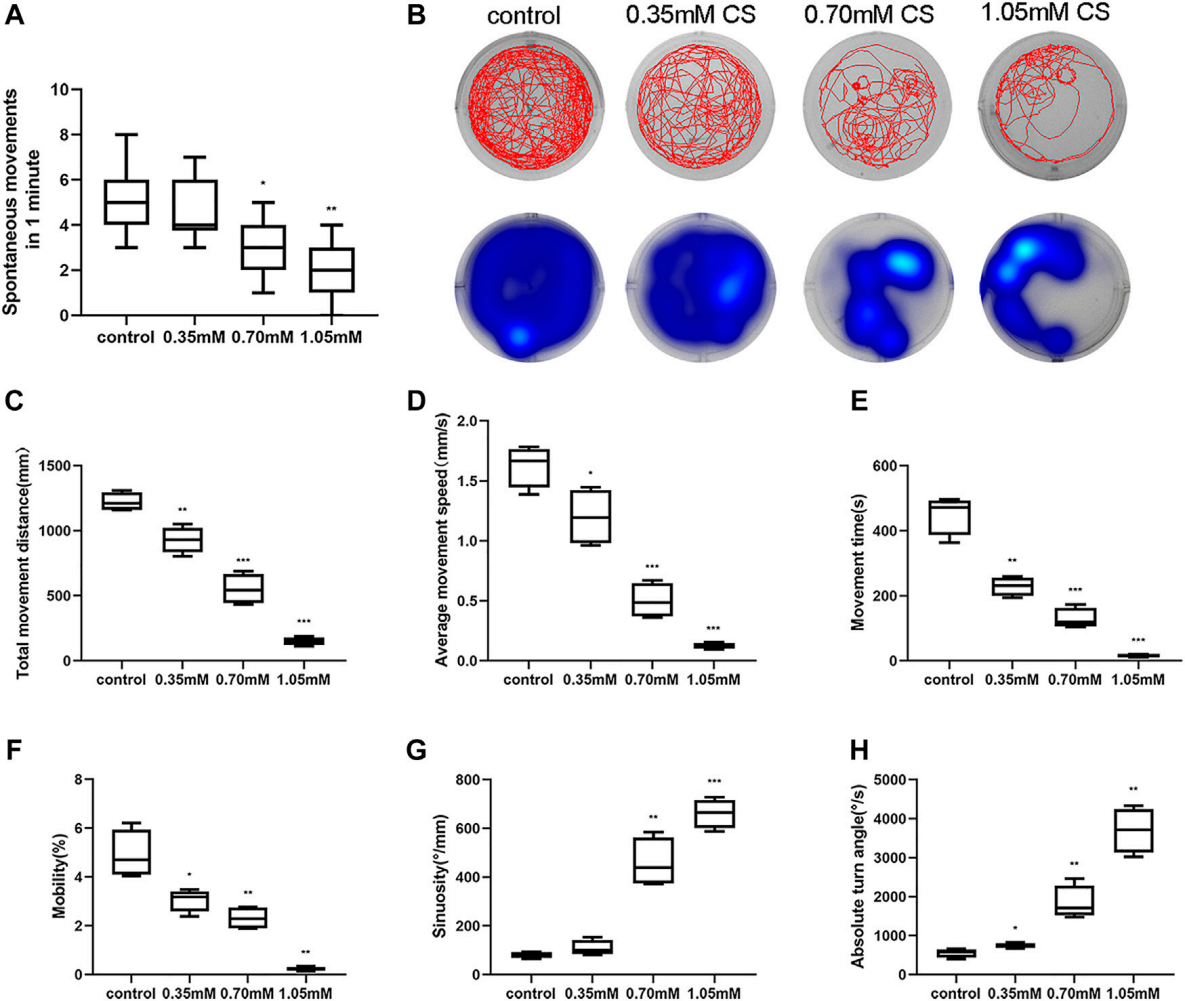
Disordering spontaneous movement and locomotor after cysteamine exposure. **(A)** Spontaneous tail coiling of 24 hpf embryos exposed to 0.35, 0.70, and 1.05 mM cysteamine. **(B)** Locomotion tracks (above) and residence time in different regions (below) of larvae exposed to 0.35, 0.70, and 1.05 mM cysteamine at 120 hpf. **(C)** Total distance. **(D)** Average speed. **(E)** Movement time. **(F)** Mobility, the frequency of high-mobile. **(G)** Sinuosity. **(H)** Absolute turn angle. The horizontal line in each box represents the average value. *p < 0.05, **p < 0.01, ***p < 0.001, mean ± S.D.

The spontaneous movement of zebrafish, which manifests in the form of spontaneous tail coiling at 24 hpf, reflects the developmental status and locomotor activity. The inhibitory effect of cysteamine on zebrafish embryonic movement at 24 hpf was concentration-dependent (*p* < 0.001, Figure 3A), which was consistent with the effect of cysteamine on zebrafish hatching rate at 24 hpf. As an indicator of skeletal developmental defects, the behavior of zebrafish larvae without deformities in each group was evaluated. The heatmaps and trajectory plots showed behavior sluggishness in the cysteamine exposure group at 120 hpf (Figure 3B). After cysteamine treatment, the total movement distance (*p* < 0.001, Figure 3C), average movement speed (*p* < 0.001, Figure 3D), movement time (*p* < 0.001, Figure 3E), and mobility (*p* < 0.001, Figure 3F) of larvae were all reduced. In contrast, the sinuosity (*p* < 0.001, Figure 3G) and absolute turn angle (*p* < 0.001, Figure 3H) increased at mounting cysteamine concentration. These data demonstrated that cysteamine exposure undermined the locomotor propensity of zebrafish larvae. This responded in part to the defective skeletal development caused by cysteamine.

### Cysteamine induced the oxidative stress accumulation

**FIGURE 4:**
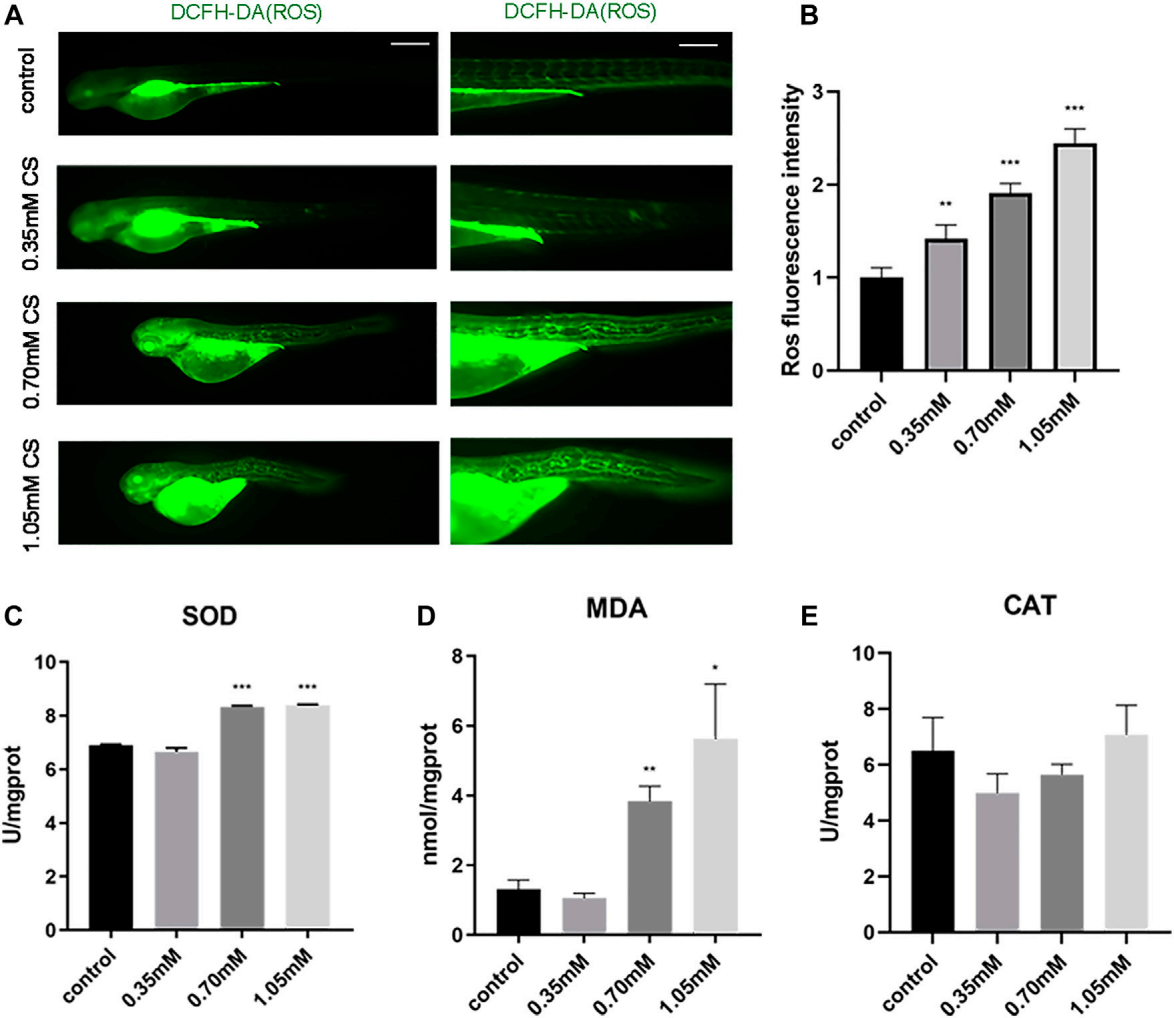
Exposure to cysteamine induced oxidative stress. **(A)** ROS staining. **(B)** ROS fluorescence intensity. **(C)** SOD activity. **(D)** MDA content. **(E)** CAT activity. Scale bars: 100 μm **(A)**. *p < 0.05, **p < 0.01, ***p < 0.001, mean ± S.D.

Bone structure destruction and dysfunction probably result from oxidative stress (Manolagas, 2010). Changes in oxidative stress were evaluated by detecting oxidative stress indicators such as SOD activity, CAT activity, MDA content, and ROS distribution. ROS fluorescence intensity was sharply enhanced in 0.35 mM (1.42 ± 0.13, *p* = 0.001), 0.70 mM (1.91 ± 0.09, *p* < 0.001) and 1.05 mM (2.45 ± 0.14, *p* < 0.001), relative to the control group (1.00 ± 0.09) (Figures 4A,B). In comparison to the control group (6.90 ± 0.02; 1.31 ± 0.22), SOD activity and MDA content considerably increased in 0.70 mM (8.33 ± 0.03, *p* < 0.001; 3.84 ± 0.35, *p* = 0.002) and 1.05 mM (8.39 ± 0.03, *p* < 0.001; 5.62 ± 1.28, *p* = 0.038), respectively (Figures 4C,D). However, CAT activity was not significantly different between the control and treated groups (Figure 4E). The results reflected a modest upward shift for oxidative stress indicators above-mentioned after cysteamine exposure, apart from CAT, which indicated that the level of oxidative stress was significantly elevated following cysteamine exposure.

### Cysteamine disordered the gene expression related to skeletal development

**FIGURE 5:**
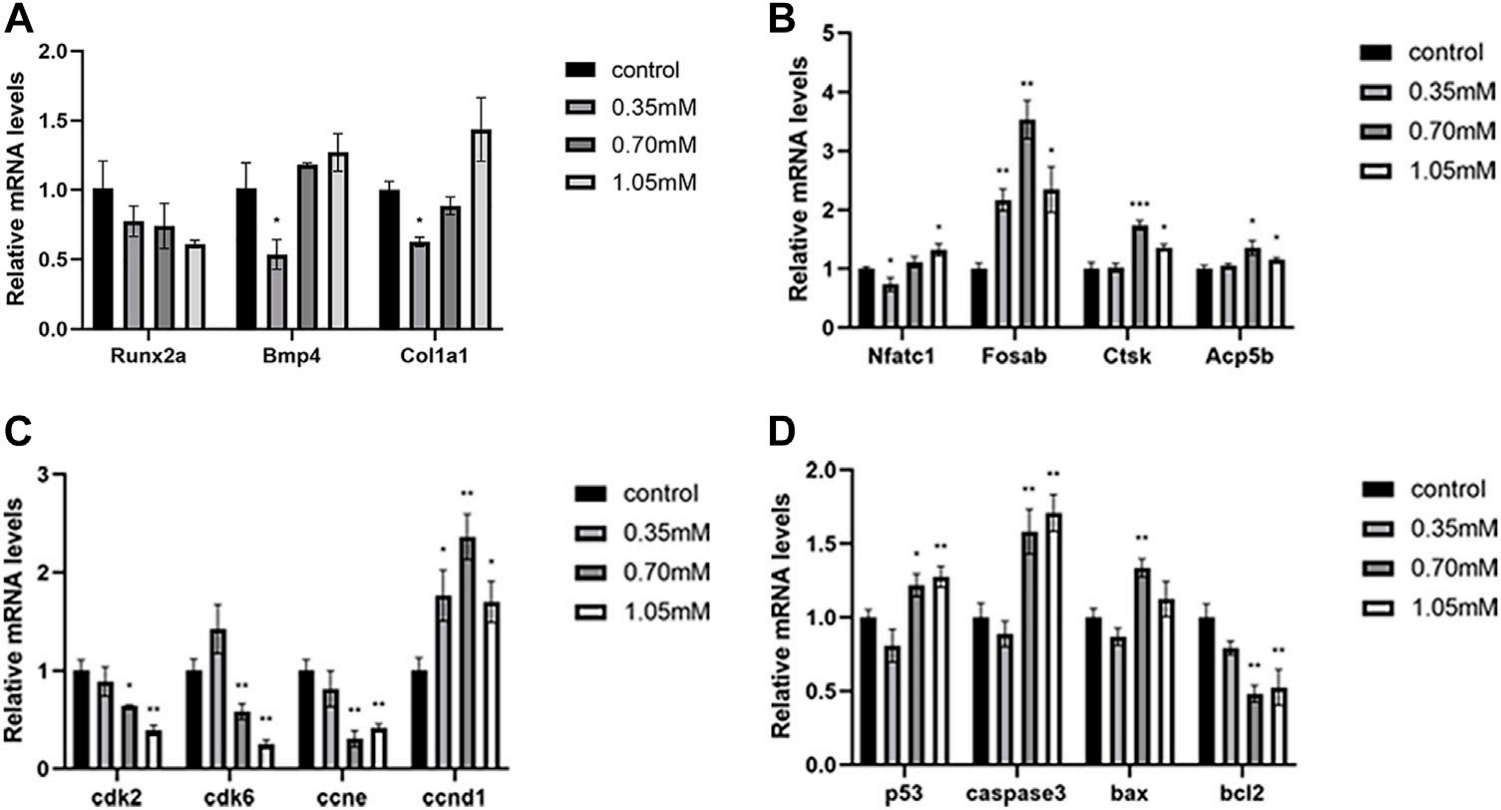
Expression analysis of related genes. **(A)** Expression analysis of osteoblast marker genes. **(B)** Expression analysis of osteoclast marker genes. **(C)** Expression analysis of apoptosis-related genes. **(D)** Expression analysis of cell cycle regulatory genes. *p < 0.05, **p < 0.01, ***p < 0.001, mean ± S.D.

To uncover the molecular mechanisms underlying the effects of cysteamine exposure on skeletal development, we detected the expression levels of osteoblast and osteoclast marker genes, cell cycle key genes, and apoptosis genes related to bone development by RT-qPCR. The results showed that cysteamine affected the mRNA expression of osteoclast marker genes (nfatc1, fosab, ctsk, and acp5b) (Figure 5B). No significant differences were observed in the mRNA levels of osteoblast marker genes (runx2a, bmp4, and col1a1) in the cysteamine group compared with the control group, except the downregulation of bmp4 and col1a1 in the lowest concentration group (Figure 5A). 

These results indicated that cysteamine influenced osteoclast differentiation during skeletal development. Furthermore, we found that the expression of key cell cycle genes ccne1, cdk2, and cdk6 was downregulated, and the expression of ccnd1 was upregulated in cysteamine exposure groups (Figure 5C). A significant upregulation for the expression of apoptotic genes (caspase3, p53, and bax) emerged after cysteamine treatment. However, the transcriptional level of bcl2, an antiapoptotic gene, experienced a drop (Figure 5D).

### The activation of Notch signal partially rescued the skeletal development defects caused by cysteamine

**FIGURE 6:**
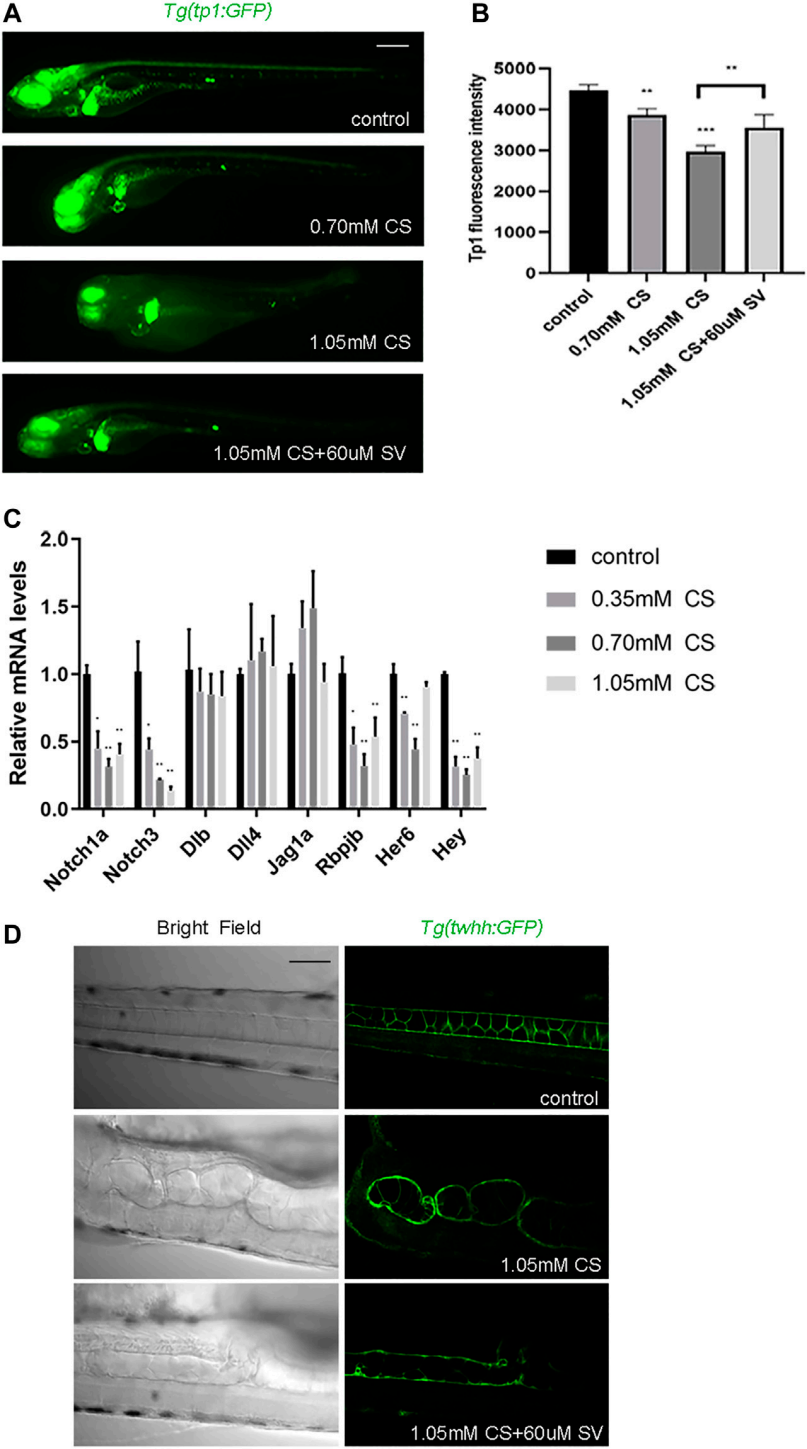
Notch signal rescued the skeletal development defects caused by cysteamine. **(A)** Tg (tp1: GFP) microscopic imaging of zebrafish larvae exposed to 0.35, 0.70, and 1.05 mM cysteamine, and 1.05 mM cysteamine with 60 μM sodium valproate at 72 hpf. **(B)** Tg (tp1: GFP) fluorescence intensity of zebrafish larvae exposed to 0.35, 0.70, and 1.05 mM cysteamine, and 1.05 mM cysteamine with 60 μM sodium valproate at 72 hpf. **(C)** Expression analysis of genes related to Notch signaling pathway. **(D)** Microscopic imaging of zebrafish *Tg* (*twhh: GFP*) larvae exposed to 1.05 mM cysteamine, and 1.05 mM cysteamine with 60 μM sodium valproate at 72 hpf. Scale bars: 100 μm **(A)**, 50 μm **(D)**. *p < 0.05, **p < 0.01, ***p < 0.001, mean ± S.D.

To confirm whether cysteamine affects Notch signaling, we initially treated *Tg* (*tp1:GFP*) transgenic zebrafish (labeled Notch signaling pathway) with cysteamine. Fluorescence microscopy imaging showed that zebrafish fluorescence decreased with cysteamine concentration increasing (*p* < 0.001, Figures 6A,B). Subsequently, the transcription level of genes related to Notch signaling was examined. The results showed that Notch signaling receptor genes (notch1a and notch3), intracellular effect molecule gene (rbpjb), and target genes (her6 and hey) were significantly downregulated with dose dependence manner (Figure 6C). While there was no significant difference in ligand genes (dlb, dll4, jag1a, and jag2b), indicating that cysteamine exposure downregulated Notch signal pathway by inhibiting the expression of Notch receptor genes. Therefore, sodium valproate (SV), an activator of Notch signaling, was used to heighten Notch signaling in zebrafish embryos. SV dramatically rescued cysteamine-induced defects in skeletal development (Figure 6D), and enhanced fluorescence of *Tg* (*tp1:GFP*) transgenic zebrafish (*p* = 0.009, Figure 6B). These results suggested that cysteamine impaired skeletal development by downregulating Notch signaling.

## Discussion

Zebrafish have facilitated a wide range of applications in pathological toxicology and ecotoxicology. We found that cysteamine severely affected zebrafish’s skeletal development and hindered the formation of the normal somite. For the first time, we discovered that cysteamine exposure significantly generated aberrant behavioral symptoms in zebrafish by behavioral analysis. The frequency of spontaneous tail coiling at 24 hpf decreased, which was related to abnormal spinal development and delayed hatching. Reduced motor performance at 120 hpf reflected skeletal maldevelopment in zebrafish similarly. Spontaneous tail coiling and locomotor behavior are important endpoints reflecting the skeletal developmental status of zebrafish embryos. According to previous reports, the occurrence of spinal deformities is accompanied by impaired spontaneous tail coiling and motor behavior (Moravec et al., 2017; Qian et al., 2020). Our results indicated that cysteamine resulted in skeletal developmental defects in zebrafish embryos. It is, to the best of our knowledge, the first example that reported the toxicological effects of cysteamine in zebrafish.

Skeletal development is accomplished by the formation and absorption of bone tissue through the mutual regulation mechanism of osteoblasts and osteoclasts (Kim and Kim, 2014). Claramunt-Taberner et al. (2018) found that cysteamine had no effects on osteoclast production, but inhibited osteoclast resorption *in vitro*; while the study by Quinaux et al. (2021) showed that cysteamine exerted inhibitory effects on osteoclast differentiation and disrupted osteoclast formation *in vitro* at high doses. In this study, cysteamine significantly downregulated the expression of osteoclast marker genes (nfatc1, fosab, ctsk, and acp5b), but had no significant effects on osteoblast marker genes (runx2, bmp4, and col1α1) apart from the downregulation of bmp4 and col1a1 in 0.35 mM group. The results indicated that cysteamine presumably regulated the differentiation and maturation of osteoclasts, stimulated the absorption of bone tissue, and ultimately affected bone development. However, this issue needs to be further explored as it may be difficult to detect the bone resorption process in the juvenile zebrafish model of choice. Witten et al. (2001) found that no acp5b (antitartaric acid phosphatase, a marker enzyme for osteoclasts) positive osteoclasts were observed in zebrafish until 20 dpf. Chen et al. (2014b) reported an absence of acp-positive osteoclasts in juvenile zebrafish sections at 10 dpf, indicating a low number and restricted distribution of osteoclasts at this time. Therefore, osteoclast involvement in zebrafish larvae up to 10 dpf was limited.

The imbalance between oxidative and antioxidant systems is another mechanism leading to skeletal toxicity by cysteamine. The level of ROS gradually ascended with the increase of cysteamine concentration. The production of ROS was widely distributed in larval zebrafish exposed to cysteamine. Compared with the control group, SOD activity increased, which can effectively remove excess oxygen free radicals. However, there was no significant difference in CAT activity. Interestingly, cysteamine, as a precursor of glutathione (GSH), is an oxidative protective agent in previous reports. This water-soluble molecule reduces oxidative damage and improves the quality of embryonic development by debasing intracellular ROS levels in mice (Aghaz et al., 2021) and pigs (Huang et al., 2021b). At the same time, it is liberally used to protect a range of organs and tissues such as the brain (Paul and Snyder, 2019), digestive tract (Liu et al., 2019), and kidney (Okamura et al., 2014) from oxidative stress under distinct conditions. In another study (unpublished), we examined the transcript expression levels of glutathione peroxidase (GPX), which indirectly responded to the effect of cysteamine on glutathione. GPX is a widespread enzyme in the organism that specifically catalyzes the reduction of glutathione to hydrogen peroxide. The results showed that 0.70 and 1.05 mM cysteamine downregulated GPX expression, implying that high concentrations of cysteamine may inhibit glutathione. These results indicated that cysteamine possibly induced oxidative stress and led to zebrafish skeletal developmental defects.

Additionally, apoptotic signals such as p53, caspase3, and bax indicated an increase in cell apoptosis. The downregulation of cell cycle key genes ccne1, cdk2, cdk6, and the upregulation of ccnd1 suggested that cell proliferation was also seriously affected by the disordered expression of these genes. According to previous publications, excessive accumulation of ROS leads to oxidative stress, affects mitochondrial function, hinders cell proliferation, and further promotes cell apoptosis (Bai et al., 2021; Wan et al., 2021). Therefore, the apoptosis of zebrafish skeletal cells exposed to cysteamine was probably mediated by oxidative stress.

Notch signaling is profoundly involved in various developmental and disease processes (Liu et al., 2022). Notch encodes receptors composed of extracellular and intracellular regions. The ligands encoded by jag1a, dlb, and dll4 interact with the extracellular domain of the Notch receptor and thereby activate the Notch signaling pathway. The Notch intracellular domain (NICD) is subsequently released and the Notch signaling transfers to the nucleus. Simultaneously, the coactivator encoded by kat2b, together with the NICD and CSL proteins encoded by recombining binding protein suppressor of hairless (rbpjb), initiates the regulation of downstream gene expression such as hey and her (Kopan and Ilagan, 2009). Abnormal activation and inhibition of the Notch pathway lead to anomalous skeletal development (Isojima and Sims, 2021). Hilton et al. (2008) observed that mutant mice lacking Notchl and Notch2 in bone marrow mesenchymal cells had bone accumulation in the bone marrow cavity at 8 weeks of age, indicating increased bone production. But by the 26th week, the trabecular mass of the mutant mice was only 10% of that of the control group, suggesting that Notch signaling probably had diverse effects on osteoblast differentiation at different developmental stages. Bai (Bai et al., 2008) co-cultured monocyte precursor cells with stromal cells expressing the Jaggedl gene (Jag1), which inhibited the promotion of osteoclastogenesis by M-CSF and RANKL, and confirmed that Notch restrained osteoclastogenesis. In this study, after zebrafish embryos were exposed to cysteamine, receptor genes (notch1a and notch3), downstream intracellular effector molecules (rbpjb), and target genes (her6 and hey) of Notch signaling were significantly downregulated, while Notch ligand genes (dlb, dll4, and jag1a) had no significant changes. Skeletal developmental defects were the result of cysteamine exposure taking into consideration the Notch signal transduction pathway inhibition with initially acting on receptors and sustained signal transduction cascade. Furthermore, we found that sodium valproate, an activator of Notch signaling (Ji et al., 2017), partially rescued the skeletal toxicity induced by cysteamine, which further proved that cysteamine caused skeletal dysplasia by inhibiting Notch signaling. According to the latest reports, N-acetylcysteine (NAC), one of the downstream metabolites of cysteamine, could suppress the Notch signaling pathway receptors Notch1, Notch2, and Notch3 in cultured cells (Zhang et al., 2016; Yao et al., 2017; Deng et al., 2019). These phenomena inferred cysteamine, as well as its derivatives, was probably involved in Notch signaling regulation jointly.

The exposure concentration of cysteamine in this study was orders of magnitude lower than the human therapeutic exposure (Liang et al., 2021). From previous reports and the results of our study, it appears that low concentrations of cysteamine have antioxidant effects and high concentrations of cysteamine may instead cause oxidative stress and affect organ development. However, studies are still needed regarding the correlation between concentrations used in zebrafish assays and those used as feed additives and drugs for pregnant women and children. Numerous studies indicate that zebrafish have a similar genetic structure to humans, sharing 70 percent of our genes. Furthermore, 84% of genes linked to human disease have a zebrafish analogue, and the zebrafish have the same primary organs and tissues as humans (Thisse and Thisse, 2008; Kwon et al., 2019; Dietrich et al., 2021). Their skeleton, blood, kidneys, and eyes have significant similarities to human systems. These findings imply that we have established a highly efficient study model, although it certainly does not reflect the human body perfectly. The molecular mechanisms by which cysteamine affects skeletal development remain to be clarified, including its principle of action with the targets and the relationship between cysteamine and other signaling pathways.

In summary, our study demonstrates that cysteamine leads to skeletal defects in zebrafish and that Notch signaling cannot be neglected in the developmentally toxic effect. Organizational abnormalities of notochord cells due to oxidative stress and apoptosis are implicated in this process.

## Data Availability

The original contributions presented in the study are included in the article/Supplementary Material, further inquiries can be directed to the corresponding authors.

## Abbreviations

CS, Cysteamine; hpf, Hour post-fertilization; hpe, Hour post-exposure; SOD, Superoxide dismutase; CAT, Catalase; MDA, Malondialdehyde; ROS, Reactive oxygen species; SV, Sodium Valproate.
