# Supplementary material for: Cysteamine affects skeletal development and impairs motor behavior in zebrafish
Source: Front Pharmacol. 2022 Aug 19;13:966710. doi: 10.3389/fphar.2022.966710 (PMC9437517; doi:10.3389/fphar.2022.966710)
Supplement: Supplementary file 1 [file DataSheet1.docx]

**Supplemental data**

**Supplemental methods**

**Distribution map of ROS**

Zebrafish embryos at 72 hpf were treated for 30 minutes at room temperature in a culture medium containing 10 mg/ml dichloro dihydro fluorescein diacetate (DFCH-DA). After being rinsed with 1×PBS, the embryos were anesthetized with 10 mg/L tricaine and examined under a fluorescence microscope (Leica, Germany). ImageJ was used to calculate the fluorescence intensity in the spinal area.

**Enzyme activity detection of SOD, CAT and MDA**

Through the xanthine and xanthine oxidase reaction system to generate superoxide anion radical, the latter oxidized hydroxylamine to form nitrite. In the role of chromogenic agent appears purple-red, the absorbance at 550nm measured by visible spectrophotometer. When the measured sample contains SOD, there is a specific inhibition of superoxide anion radicals, so that the formation of nitrite is reduced. The absorbance value of the colorimetric measurement tube is lower than the absorbance value of the control tube, and the difference between the two can be converted to measure the SOD activity in the sample. The amount of SOD corresponding to 50% SOD inhibition per ml of reaction solution is defined as one SOD activity unit (U).

The reaction of CAT to decompose H_2_O_2_ can be rapidly suspended by adding ammonium molybdate, and the remaining H_2_O_2_ interacts with ammonium molybdate to produce a yellowish complex. The absorbance (at 405nm) change before and after the reaction can be measured to calculate the CAT activity.

MDA in peroxidized lipid degradation can be condensed with thiobarbituric acid to form a red product. The absorbance at 532 nm can be measured to calculate the CAT activity.
